# Gene Networks Driving Genetic Variation in Milk and Cheese-Making Traits of Spanish Assaf Sheep

**DOI:** 10.3390/genes11070715

**Published:** 2020-06-27

**Authors:** Héctor Marina, Antonio Reverter, Beatriz Gutiérrez-Gil, Pâmela Almeida Alexandre, Laercio R. Porto-Neto, Aroa Suárez-Vega, Yutao Li, Cristina Esteban-Blanco, Juan-José Arranz

**Affiliations:** 1 Departamento de Producción Animal, Facultad de Veterinaria, Universidad de León, Campus de Vegazana s/n, 24071 León, Spain; hmarg@unileon.es (H.M.); beatriz.gutierrez@unileon.es (B.G.-G.); asuav@unileon.es (A.S.-V.); cestb@unileon.es (C.E.-B.); 2 CSIRO Agriculture and Food, Queensland Bioscience Precinct, 306 Carmody Rd., St Lucia, Brisbane, Queensland 4067, Australia; toni.reverter-gomez@csiro.au (A.R.); Pamela.Alexandre@csiro.au (P.A.A.); laercio.portoneto@csiro.au (L.R.P.-N.); Yutao.Li@csiro.au (Y.L.)

**Keywords:** dairy sheep, milk coagulation properties, meta-analysis, GWAS, stepwise analysis, pleiotropy, linkage disequilibrium

## Abstract

Most of the milk produced by sheep is used for the production of high-quality cheese. Consequently, traits related to milk coagulation properties and cheese yield are economically important to the Spanish dairy industry. The present study aims to identify candidate genes and their regulators related to 14 milk and cheese-making traits and to develop a low-density panel of markers that could be used to predict an individual’s genetic potential for cheese-making efficiency. In this study, we performed a combination of the classical genome-wide association study (GWAS) with a stepwise regression method and a pleiotropy analysis to determine the best combination of the variants located within the confidence intervals of the potential candidate genes that may explain the greatest genetic variance for milk and cheese-making traits. Two gene networks related to milk and cheese-making traits were created using the genomic relationship matrices built through a stepwise multiple regression approach. Several co-associated genes in these networks are involved in biological processes previously found to be associated with milk synthesis and cheese-making efficiency. The methodology applied in this study enabled the selection of a co-association network comprised of 374 variants located in the surrounding of genes showing a potential influence on milk synthesis and cheese-making efficiency.

## 1. Introduction

Sheep milk production is highly important in Mediterranean and Middle Eastern countries. Spain holds one of the highest dairy sheep livestock counts in Europe [1], and almost all the sheep milk produced is used for the production of high-quality cheese [2]. Milk composition has a strong influence on the technological and organoleptic characteristics of dairy products [3]. Sheep milk properties enable sheep cheeses to have better sensory characteristics than cheeses from goat and cow milk [4].

Breed selection schemes in dairy sheep are generally focused on milk yield and fat and protein contents. Therefore, genetic parameters for these traits have been widely studied [5,6,7]. However, few genetic studies have investigated the genetic component involved in the cheese-making process through the analysis of milk traits (milk yield and composition) and cheese-making traits (milk coagulation properties (MCP) and cheese yield-related traits) [8,9,10]. Cheese-making traits are difficult to measure in routine integration into classical breeding programmes, therefore the identification of genetic markers associated with these genes may be of high relevance to the sheep dairy industry.

To elucidate the complex genetic architecture underlying milk traits, several research approaches have been performed. Previous studies have focused on the evaluation of polymorphisms in ovine major milk proteins (caseins and whey proteins) and genes related to the fat synthesis of milk. Some of these polymorphisms have been associated with milk yield, protein and fat milk contents and milk technological properties [3,11,12,13]. Complex traits, such as milk and cheese-making traits, are assumed to be influenced by many genomic regions. In this sense, the availability of genome-wide Single Nucleotide Polymorphisms (SNPs) panels has enabled the identification of genomic regions associated with complex traits in many cases by applying the genome-wide association study (GWAS) approach. Detailed information about the genomic regions or quantitative trait loci (QTLs) influencing traits of interest in dairy sheep identified by association–based studies can be found in the SheepQTLdb [14]. Despite the considerable advantages of the GWAS approach in the identification of genomic regions associated with these traits, we need to consider that, for complex traits, it is difficult to devise experimental designs with adequate power to identify genes that contribute to the genetic variance of these traits [15]. Some specific statistical procedures, such as stepwise regression, may help to overcome this power limitation. In addition, integrated approaches, such as those based on partial correlation and information theory (PCIT) [16], have attempted to enrich GWAS analyses with information from other sources, providing useful alternatives for characterising genes and gene networks associated with complex traits [17,18]. Generating knowledge on these gene networks may help to elucidate the genetic architecture of complex traits and thus develop genomic tools with predictive value for such traits.

In the present study, by using a custom 50K chip integrating SNPs identified in a previous study by our group investigating the variability of the sheep milk transcriptome [19], we applied a stepwise procedure in combination with classical GWAS, co-association network (PCIT) and pleiotropy analyses to decipher the genetic architecture of 14 milk and cheese-making traits measured in a commercial population of Assaf. The principal aim of this stepwise analysis is the identification of gene networks (candidate genes and their potential regulators) and biological processes implicated in milk synthesis and cheese-making production efficiency. The results described in this report have enabled us to select a panel of markers that could be used as predictors of an individual’s genetic potential for milk and cheese-making. This study may provide a practical and cost-effective solution for the genetic improvement of these economically important traits in the studied population.

## 2. Materials and Methods 

### 2.1. Animals and Phenotypes

A total of 1039 ewes of the Spanish Assaf commercial population were analysed in this study. A milk sample was collected from each ewe in the morning milking following the procedure described by Sánchez-Mayor et al. [10]. Each sample was analysed to determine seven milk traits and six cheese-making traits.

The milk traits included five traits related to milk production, that is, milk yield (MY, kilograms), fat percentage (FP, %), lactose percentage (LP, %), protein percentage (PP, %), and dry milk extract (DE, %), and two related to milk functional traits, that is, somatic cell count (SCC, number of cells per mL) and the pH of milk.

To determine the individual cheese-making efficiency, we measured two groups of traits, milk coagulation properties (MCP) and traits related to cheese yield. The MCP includes the time between rennet addition and the formation of the curd, also known as rennet clotting time (RCT, min); the time necessary for the curd to reach 20 mm or curd-firming time (K20, min); and the curd firmness at 30 and 60 min after rennet addition (A30 and A60, mm). In addition, we calculated the logarithm of the ratio RCT/A60, which has been declared as an indicator of coagulation efficiency [20]. Moreover, two traits related to the cheese yield were recorded: the laboratory cheese yield (ILCY, g/10 mL of milk) was obtained following Othmane et al. [9,21], and the individual laboratory dried curd yield (ILDCY) was estimated by maintaining the drained curds in an oven at 100 °C for 24 h (ILDCY, g/10 mL).

### 2.2. Ethics Committee Approval

As the animals have been sampled within the routine practices carried out on commercial farms, and after consultation with the Research Ethics Committee of the University of León, we have been advised that formal ethical approval is not required under these circumstances.

### 2.3. Design of a Custom Ovine SNP Array and Genotypes

An ovine custom 58,983 SNP array was designed by including 3173 variants selected from a previous study of our research group characterising the genetic variation within the milk somatic cell transcriptome of lactating sheep [19]; these variants have been added to the custom chip routinely used by the Assaf and Churra breeders’ association in their genomic selection programmes. The selection criteria for the inclusion of transcriptome markers were as follows: from all the SNPs identified in the milk somatic cell transcriptome of the Churra and Assaf breeds, 91,683 with an MAF > 0.125 in both breeds were selected. Of these SNPs, 1161 located at genes involved in milk protein and fat synthesis, as well as at candidate genes from regions with QTLs for milk traits, were chosen. Finally, 2130 SNPs located in the genomic regions with low coverage were added to ensure a uniform distribution of approximately one functional marker per megabase of the ovine autosomal genome.

Blood samples from the 1039 ewes were collected for DNA extraction and subsequent genotyping with the 50K SNP array. Raw genotypic data were subjected to quality control filtering. Those markers with a minor allele frequency (MAF) higher than 5% and genotype frequencies in Hardy–Weinberg equilibrium (HWE) [*p*-value > 0.05] were included in the following analyses. Markers remaining after quality filtering were used to construct a genomic relationship matrix (GRM) [22] among the 1039 samples analysed. The GRM was represented following the pedigromics pipeline [23] to evaluate the population structure of the ewes used for the analyses.

### 2.4. Genetic Parameter Estimation

To estimate the genomic breeding values (GEBV) and the variance components of each trait, we performed an average-information restricted maximum likelihood (AIREML) algorithm in a univariate mixed model analysis for each trait included in this study using the BLUPF90 family of programmes [24]. The following mixed model was used to fit the fixed and random effects simultaneously:

Y = Xb + Za + e

where Y is the vector of phenotypes, X is the incidence matrix of fixed effects, b is the vector of fixed effects, including days in milk (DIM) as a covariate and the age at parturition combined with the number of births (AGE_NB: 18 levels), the flock test day (FTD: 12 levels) and the number of born lambs (NBL: 2 levels, one or two lambs), as factors. Finally, Z is the incidence matrix that relates animals to traits, a is the vector of random SNP additive effects, and e is the vector of residual effects. In the model, we assume that the random effects a and e are normally distributed with zero mean and variance GRMσa2 and I(nxn)σe2, respectively. In this instance, σa2 and σe2 are additive genetic and error variances, I is an identity matrix, and n is the number of animals. In addition, a bivariate analysis was performed to investigate the phenotypic and genotypic correlations among the milk and cheese-making traits, and their genetic architecture was shown by the weighted average of heritability by its standard error (h2 SNP). The errors of the genetic correlations were estimated from the weighted average of standard error by the heritability of both traits.

Hence, an analysis of variance (ANOVA) was performed using a multifactorial least square regression model through the SAS program (SAS Inst. Inc., Cary, NC, USA). ANOVA was applied to identify the effect of the fixed factors included in this model on the traits under investigation and the possible influence of the milk traits on the coagulation success factor (defined by a categorical factor, such as coagulating and non-coagulating milk).

### 2.5. Genome-Wide Association Study and Pleiotropy Analysis

To evaluate the effect of the total SNPs retained after quality control, a GWAS was performed for the 14 traits included in this study using our own Fortran source code according to Garrick et al. [25]. Previously, in order to perform the pleiotropy and stepwise regression forward selection analysis, we selected the SNPs located within a gene or a 20-kb distance of a known gene. Therefore, each of the SNPs used in the subsequent analyses was representative of an annotated gene in the sheep Oar v3.1 genome assembly. The gene annotation was obtained from Biomart software [26]. With the 12,426 variants located within the confidence interval of a gene, we performed a pleiotropy analysis using a multi-trait statistic to determine the effect of each SNP across the milk and cheese-making traits following the Bolormaa et al. [27] procedure. The results obtained by the GWAS and pleiotropy analyses were used for the selection and representation of the genes used in the co-association networks, as outlined below.

### 2.6. Stepwise Multiple Regression Analysis

**Figure 1:**
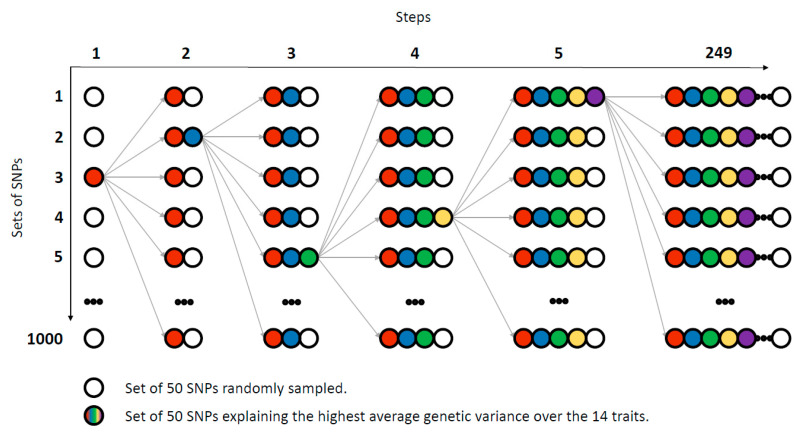
Graphic description of the stepwise multiple regression analysis. This figure graphically represents the selection method applied to the stepwise regression analysis, which was based on adding 50 new SNPs each step of the analysis, until all the variants were included. Each circle represents a set of 50 randomly selected SNPs. The colours represent the groups of SNPs which explained the highest average genetic variance over the 14 traits selected in the subsequent steps, first step red, second blue, and so on).

Subsequently, a stepwise multiple regression analysis was also performed over the 12,426 markers located within the confidence interval of a gene. At each step, 1000 sets of 50 SNPs were randomly sampled. The set explaining the highest average genetic variance over the 14 traits was included in the regression analysis. Therefore, each step added 50 new SNPs to the regression analysis until all the variants were included (Figure 1). The SNPs selected in each round were automatically assigned to the following steps, to which another set of 50 SNPs was added following the same searching scheme. The analysis was considered finished when all the variants were included.

### 2.7. Gene Co-association Network and Functional Enrichment Analysis

To identify the significant co-associations between the genes selected from the stepwise analysis, we employed the partial correlations and information theory (PCIT) algorithm developed by Reverter and Chan [16] using the effect of each SNP on the traits under study and visualised the gene to gene association network with Cytoscape 2.8.3 [28]. The PCIT has been used to ascertain the significance of the correlations between gene pair through the comparison whit any other gene in the dataset. As detailed above, each of the SNPs analysed in the GWAS, pleiotropy and stepwise analyses was considered representative of a gene. Hence, co-association networks were created using the representative gene of each SNP. Two co-association networks were constructed with different gene subsets. The first network was built using the set of genes (550 genes) explaining more than 95% of the genetic variance, while the second network was created using the set of genes (5450 genes) explaining the highest genetic variance achieved in the analysis. The transcription and co-transcription factors of each set of genes were identified through AnimalTFDB 3.0 [29]. The final networks were composed of the genes matching the following criteria: (i) to be significantly co-associated by the PCIT algorithm with at least five (first gene-set selected) or 50 (second gene-set) transcription and co-transcription factors and (ii) to be significantly associated (*p* < 0.05) in the GWAS analysis with at least one of the traits under study.

The genes of both networks were classified according to the group of traits to which they were associated (milk traits, cheese-making traits or both) and the kind of gene (transcription factor, co-transcription factor or other genes). The relationship among gene groups was visualised through the spring embedded algorithm to illustrate them by the radiality. The coordinates of the genomic regions containing QTLs related to milk traits and cheese-making traits were downloaded from SheepQTLdb (http://www.animalgenome.org/cgi-bin/QTLdb/OA/search) [14]. The Ensembl database [26] was used to determine the orthologous human gene IDs (Homo sapiens) in the two gene sets selected. Finally, to obtain the gene ontology (GO) terms related to the genes of both networks (biological process), two bioinformatic tools were used, the WEB-based Gene SeT AnaLysis Toolkit (WebGestalt) [30] and the Panther classification system [31].

### 2.8. Genetic Variance Explained Randomly and Relationship Matrix

To validate the percentage of genetic variance explained by our two co-association networks, we contrasted these networks to 1000 random selections of SNPs located within genes of the same size as the gene sets selected. This procedure enabled us to compare the genetic variance explained by the SNPs selected in this study, in contrast to the genetic variance explained by a random selection of SNPs.

In addition, following the pedigromics pipeline [23], both gene sets were used to create two different GRMs to visualise how the relationship among the individuals included in the study changes according to the set of SNPs selected. The samples were ranked using a z-score designed to summarise the breeding value across the 14 milk and cheese-making traits analysed. The *z*-score was computed after summing the GEBV values of each trait obtained by the univariate mixed model correction explained above. The scale of the GEBV values was inverted in those traits related to time values (K20, RCT and RCT/A60), the SCC and the pH to obtain a z-score whose higher values reflect higher genetic values of cheese-making traits.

## 3. Results

**Figure 2:**
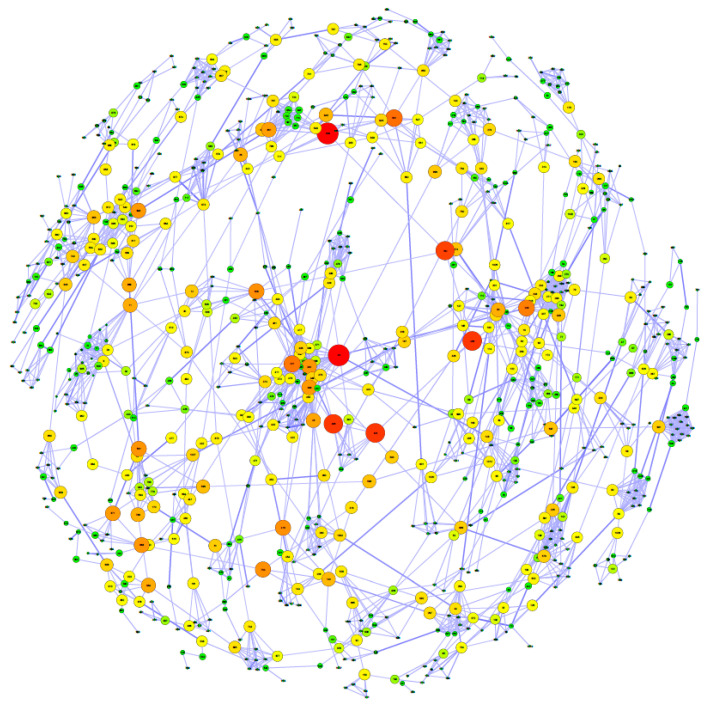
Pedigromics. This figure shows the relationship (>0.2) among the individuals included in this study. Each node represents one animal from the population; those animals not related to the main population were filtered. The colour and the size of the nodes are based on the betweenness coefficient, on a green to a red colour scale, with the higher values represented by a large size and red colour. The width of the connection line (edge) depends on the values of the off-diagonal elements in the GRM.

The population structure of the 1039 ewes included in the study was represented using the GRM created with the 43,882 SNPs remaining after quality control filtering (Figure 2). To simplify the network, only genomic relationships higher than 0.20 were represented. We can observe that there are several small family groups linked by their ancestors and descendants, showing a non-structured population. Neither inbreeding, heterosis or dominance effects have shown a significant effect (depression or boosting) on any of the traits analysed in this study.

### 3.1. Genetic Parameters of the Analysed Phenotypes

**Figure 3:**
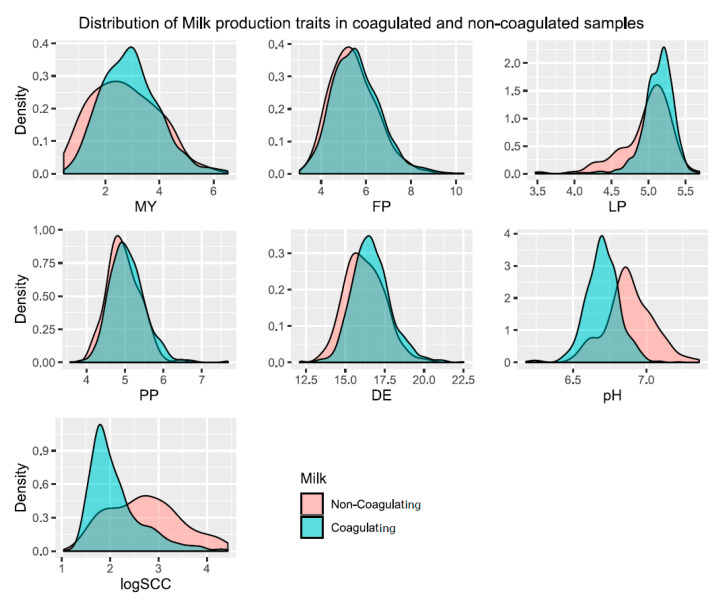
Distribution of the milk trait values in relation to the coagulation success factor. This figure displays the distribution of values of the seven milk traits considered in this study in contrast to the coagulation success factor (coagulating or non-coagulating milk). Represented traits are milk yield (MY), protein percentage (PP), fat percentage (FP), lactose percentage (LP), dry extract (DE), pH, and the decimal logarithm of somatic cell counts (logSCC).

Of the 1039 samples included in the study, 131 samples did not coagulate successfully within 60 min after the addition of the clotting enzyme; therefore, they had no values for the cheese-making traits and were declared missing values for the subsequent analyses. The multifactorial ANOVA confirmed the significance of the fixed effects included in the model (AGE_NB, DIM, FTD and NBL) and the phenotypic corrected variance explained by the model. Moreover, ANOVA revealed a significant association (*p*-value < 0.001) between four milk traits (MY, LP, logSCC and pH) and coagulation success (Table 1). The distribution of the milk trait values concerning the coagulation success factor is represented in Figure 3. The ANOVA also showed a significant effect of DIM on two of the milk traits (MY and pH) and three of the cheese-making traits (A30, RCT/A60 and ILDCY) (Table 2).

The phenotypic average, additive genetic variances and heritability estimates for the 14 traits related to milk production and cheese-making traits are given in Table 3. The heritabilities for milk traits ranged from low (0.05 for FP and logSCC) to moderate values (>0.30 for pH and PP), with pH having the highest heritability (0.37). On the other hand, the cheese-making traits displayed moderate estimates of heritability, ranging from 0.17 (A60 and logRCT/A60) to 0.33 (logK20 and ILCY). Table 4 shows the average heritability weighted by the standard error of each trait and the phenotypic and genomic correlations. Generally, the phenotypic correlations among the 14 traits were low, except for some traits that showed absolute correlation values higher than 0.80 (A30-RCT, A60-logRCT/A60, DE-FP, ILCDY-ILCY and logRCT/A60-RCT). Conversely, the genotypic correlations showed strong values within and between the milk traits and cheese-making traits (Table 4). Some of the milk traits were strongly genetically correlated (exhibiting absolute values higher than 0.80). Notably, some genetic correlations were found among the cheese-making traits, such as those between pH and ILCY (−0.89), DE and ILCDY (0.99), FP with either A60 (−0.99), ILCY (0.99) and ILCDY (0.99), and between logSCC and A60 (0.83).

### 3.2. Stepwise Analysis, Gene Co-association Network and Potential Regulators

The objective of these analyses was to obtain a set of SNPs colocalised in the confidence interval of a gene (20 kb), which could explain a high proportion of the genetic variance for the 14 traits under study. From the total of markers that passed quality control (43882 SNPs), a total of 12426 SNPs were mapped within the confidence interval of a gene (see Table S1). The global distributions of the genetic variance explained in each step of the stepwise analysis (1000 randomly sampled rounds per step) are shown in Figure S1. The addition of SNPs at each step of the analysis allowed us to increase the genetic variance explained by the set. Once the maximum genetic variance explained was reached, the addition of new markers decreased the genetic variance that could be explained by the set of SNPs until it reached the variance explained by the whole set of SNPs that passed the quality control. The correlations between the off-diagonal elements of each GRM based on the subsets of genes selected along with the stepwise procedure and the off-diagonal elements of the GRM with all genes are represented in Figure S2. This figure illustrates how the relationship among individuals changes by adding new SNPs in each step of the stepwise procedure.

From the stepwise analyses, we selected two subsets of genes that explained (1) on average more than 95% of the genetic variance for all the traits and (2) the highest genetic variance explained by the analysis. The first subset of genes was found in the 11th step of the stepwise analysis, where 550 genes were selected. The highest genetic variance was explained in the 109th step, corresponding to a subset of 5450 genes, which could explain 2.54 times more genetic variance than that explained by all SNPs.

**Figure 4:**
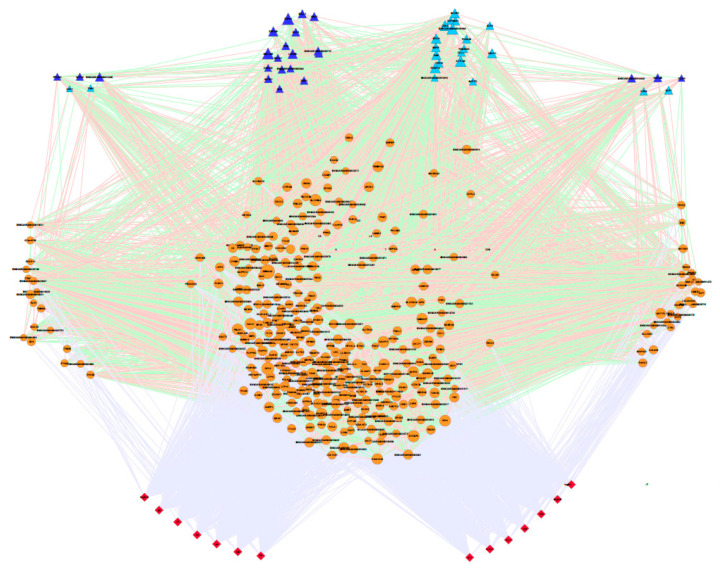
Co-association network between the 374 genes selected by the stepwise procedure. This network includes all the transcription (TF: depicted in dark blue) and co-transcription factors (CF: described in light blue) and the rest of genes (painted in orange), together with the 14 traits analysed (depicted in red). The significant co-associations between the pair of genes and the significant association between a gene and a trait were represented with an edge (Positive co-associations: green, Negative co-associations: red; GWAS association: grey). The size of the nodes was based on the pleiotropic effect of the gene on the traits under study. This co-association network explained a total of 76% of the genetic variance.

**Figure 5:**
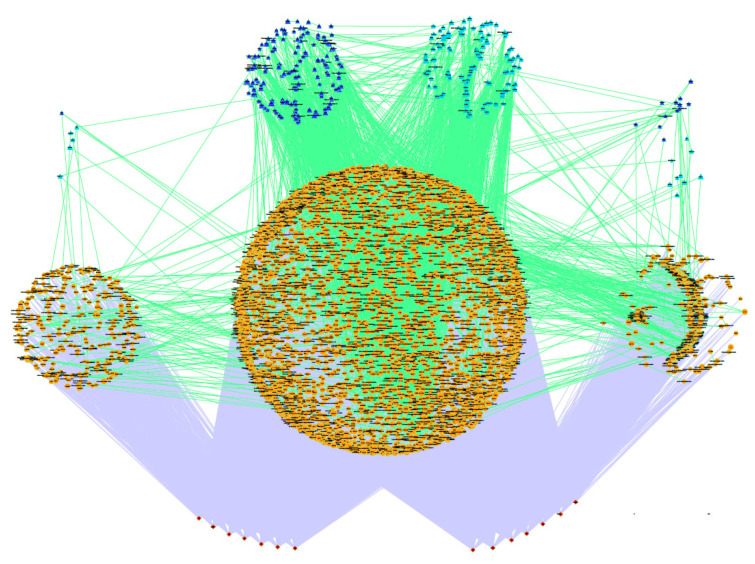
Co-association network between the 4585 genes selected by the stepwise procedure. This network includes all the transcription (TF: depicted in dark blue) and co-transcription factors (CF: depicted in light blue) and the rest of genes (painted in orange), together with the 14 traits analysed (described in red). The significant co-associations between the pair of genes and the significant association between a gene and trait were represented with an edge (Positive co-associations: green; GWAS association: Grey). The size of the nodes was based on the pleiotropic effect of the gene on the traits under study. This co-association network explained 2.4 times the total genetic variance.

After filtering the two subsets considering the significant association of genes/SNPs in the GWAS analysis with at least one trait and their co-association with transcription and co-transcription factors, 374 genes (from the 550-gene set) and 4586 genes (from the 5450-gene set) were retained for the co-association network analysis (see Table S2). To simplify the gene networks, only the significant gene-gene co-association values obtained through the PCIT algorithm higher than 0.80 (550 genes) and 0.95 (5450 genes) and the significant gene–trait association (*p* < 0.05) were represented, as shown in Figure 4 and Figure 5, respectively. The co-association networks were clustered according to whether they were associated with milk traits, cheese-making traits or both groups. In this way, we reclassified these three clusters by grouping the transcription factors (TF), co-transcription factors (CF) and the rest of the genes. Consequently, networks allow exploration of the co-association between all genes (TF, CF and the rest of the genes) and the association of these genes with the 14 traits under study.

The average genetic variance explained by the markers in the two co-associated networks constructed, in comparison with the total genetic variance explained by all filtered SNPs, was 76% when using 374 genes and 1.5 times when using 4586 genes. However, the random sampling of 374 and 4586 SNPs was respectively able to explain, on average, 15% and 79% of the average genetic variance considering all traits under study, which highlights the importance of the SNPs selected for both co-associated networks to explain the genetic variance of the traits under study. Additionally, within the 12,426 SNPs mapped in the confidence interval of a gene, 6112 SNPs were located within genes previously identified as expressed in the lactating mammary gland by Suárez-Vega et al. [19] (see Table S1). This set of SNPs within genes expressed in the milk transcriptome, one variant per gene, could explain for each trait, on average, 97% of the total genetic variance explained by all high-quality SNPs considered in the study.

Finally, we examined how the selected gene networks affected the relationship among the sampled ewes. The GRM created based on both gene co-association networks can be seen in Figure S3. The *z*-score values, designed to summarise the GEBVs corresponding to the milk and cheese-making traits, showed a normal distribution with zero mean and SD of 3.73. Animals with higher values are related to higher milk quality and cheese-making efficiency. The pedigromics based on the 374 genes selected in the first co-association network showed a highly correlated population in contrast with the pedigromics based on the 4586 genes selected in the second co-association network, where the animals were arranged in small groups with similar z-score values.

### 3.3. Identification of Enriched Gene Set

The enrichment analysis was performed on the two co-association networks, considering the orthologous human gene IDs: 304 (from the 374 genes selected) and 3601 genes (from the 4586 genes selected), respectively. In total, 264 and 3112 genes were related to at least one biological process through the Panther classification system, and each gene was associated with 5.30 and 1.92 gene ontology (GO) terms on average, respectively, for each of the co-association networks. Within the two gene networks, 194 and 2275 genes, respectively, were previously identified as being expressed in the lactating mammary gland transcriptome of Assaf and Churra Spanish sheep (see Table S2).

The network derived from the co-association analysis obtained by partial correlations and information theory (PCIT) in the 11th step contained 374 nodes (i.e., Genes) and 16,475 edges (i.e., significant associations and co-association correlations). Of these genes, 23 were annotated as TF and 22 as CF, and they together were considered potential regulators of the network (see Table S2). The main families of TFs that constitute the network were zinc finger families (zf-C2H2 zf-MIZ), homeobox and ETS (E26 transformation specific), and the key families of CFs were elongator acetyltransferase (ELP) and cyclin. The 45 regulators were involved in the following biological processes: transcription by RNA polymerase II (GO:0006366), regulation of cell cycle (GO:0007049), cell differentiation (GO:0030154) and cell proliferation (GO:0008283), multicellular organism development (GO:0007275), response to lipid (GO:0033993), lipid storage (GO:0019915), fatty acid metabolic process (GO:0006631), intracellular protein transport (GO:0006886), response to stimulus (GO:0050896), response to stress (GO:0006950) and circadian rhythm (GO:0007623). In general, genes that conform to the network were involved in 1611 biological processes, which were generally related to basal and essential cell functions, and biological processes, which could directly influence milk quality and cheese-making traits. Briefly, some of these significant biological process terms were calcium ion transmembrane transport (GO:0070588), fatty acid β-oxidation (GO:0006635), fatty acid metabolic process (GO:0006631), fatty acid transport (GO:0015908), glucose transmembrane transport (GO:1904659), phospholipid transport (GO:0015914), and positive regulation of prolactin secretion (GO:1902722) (see Table S3). A total of 10 QTLs were identified by association studies within the regions where these 374 genes were located. Three of the TFs identified through the stepwise analysis (*MECOM*, *ZFPM1*, *ZNF250*) were located in regions where six QTLs related to milk yield, milk fat and protein percentage have been previously described. The gene *EGFLAM* was also located in a region where four QTLs related to somatic cell count, bacterial milk count and clinical mastitis were described (see Table S4).

On the other hand, the network derived from the co-association analysis obtained in the 109th step of the stepwise analysis gathered 4586 nodes and 25,688 significant co-association and association links. The maximum proportion of explained genetic variation was achieved after continuing the selection of variants for 99 more steps than the network described previously. After networks were filtered, a total of 274 TF and 223 CF were considered potential regulators of the network, as indicated in Table S2. The most predominant TF families found were zf-C2H2 and homeobox in addition to bHLH (basic helix–loop–helix) and HMG (high mobility group). The main families of CF were cyclin, nuclear and lysine. According to the enrichment analysis, this group composed of 497 regulators is involved in several biological processes, the most common of which were transcription by RNA polymerase II (GO:0006366; GO:0006357; GO:0045944, GO:0000122), cell cycle (GO:0007049), cell differentiation (GO:0030154), transcription of DNA templated (GO:0006351; GO:0006355), response to lipid (GO:0033993), multicellular organism development (GO:0007275) and hormone-mediated signalling pathway (GO:0009755) (see Table S2). At the level of the entire gene co-association network, several biological processes were significantly associated. Among these processes, we highlight those that could affect traits under study, such as cell morphogenesis (GO:0030030), cell projection organisation (GO:0000902), cell differentiation (GO:0000904), cell development (GO:0060284), cellular response to stress (GO:0033554), cellular protein localisation (GO:0034613), homeostatic process (GO:0042592), ion transport (GO:0006811), ion transmembrane transport (GO:0034220), positive regulation of molecular function (GO:0044093), protein phosphorylation (GO:0006468) and regulation of phosphorylation (GO:0042325) (see Table S3). Considering the 4586 genes selected for the second network by the stepwise analysis, a total of 115 QTLs were identified in the confidence interval of 63 gene regions. These QTLs were associated with milk yield, milk fat, protein, lactose and casein percentage, bacterial milk count, somatic cell count and curd firming time, as summarised in Table S4.

### 3.4. Pleiotropy of the Selected Genes

The pleiotropic effect was quantified in every SNP located within the confidence interval from a gene through the effect obtained by the 14 GWAS that were performed, that is, one for each analysed trait. The pleiotropic values showed a normal distribution with a mean of 49.47 and standard deviation of 19.04. In general, markers with a high pleiotropic value were selected by the stepwise procedure in early rounds. The highest pleiotropic effects in the two gene sets were found in the group of genes related to both groups of traits (milk traits and cheese-making traits), as would be expected, and the average of a pleiotropic effect of the CF was higher than the rest of the genes in both gene sets selected. Specifically, the highest pleiotropic effect was found for the gene Semaphorin 4A (*SEMA4A*), primarily related to the following biological processes: animal organ development (GO:0048513), regulation of cell growth (GO:0030308), cell migration (GO:0030335), cell size (GO:0008361) and tissue development (GO:0009888).

## 4. Discussion

Almost all the milk produced from Spanish Assaf ewes is used for cheese manufacturing. Therefore, cheese-making traits could be used as selection criteria in dairy sheep breeding programmes. However, the routine measurement of milk traits is simpler and less expensive than that for cheese-making traits, especially at the individual animal level. Since milk traits are already considered selection criteria in genomic selection programmes of dairy sheep, previous studies have focused on identifying the relationship between milk’s physicochemical composition parameters and cheese-making variables [10,15,20]. Furthermore, concerning the milk coagulation properties and cheese yield in the Assaf breed, the genetic parameters of these traits have been adequately discussed in a recent paper by our group [10]. In this study, we analysed seven milk traits and seven cheese-making traits through a stepwise procedure in combination with classical GWAS, pleiotropy and co-association analyses. Our main aim was to identify SNPs located within a confidence interval of genes that are relevant to the traits considered, which could be used in genomic selection programmes applied in dairy sheep.

Regarding the results of the multifactorial ANOVA (Table 1), our results regarding the most important role of pH on milk coagulation efficiency followed by the effect of SCC agree with previously reported studies [20]. On the other hand, our analysis related a high initial milk pH measurement, low SCC and low lactose content to inefficiency in the coagulation process (Figure 3), which is in agreement with previous reports [2,20,32]. In addition, we found an influence of the DIM on the milk and cheese-making traits (Table 2) according to Jaramillo et al. [2], who described the variation of the renneting variables and physicochemical milk composition during lactation in sheep.

The high correlations found among the two families of traits that were analysed (Table 4) support the possibility of using these correlations to predict the GEBV from cheese-making traits from milk phenotypes, whose sampling is implemented in the official milk recording system, and the genotypes of the SNP chip. To this end, a stepwise analysis strategy has been applied to obtain the minimum number of SNPs that can explain the maximum genetic variance for both types of traits.

The stepwise regression forward selection method generates a GRM in each step, attempting to capture as much additive genetic variance as possible for each trait. The variation of the genomic relationship between the animals of the population (Figure S2) enabled us to reach the maximum of the genetic variance explained through the design of an idealised pedigree, achieved in the 109th step of the stepwise method, where the animals were arranged in small groups with similar z-score values (Figure S3). Hence, the z-score estimated here summarises the cheese-making aptitude based on the 14 traits analysed in this study.

The genetic component is one of the factors influencing cheese production; therefore, elucidating the genomic regions related to milk and cheese-making traits might help to elucidate the genetic background underlying cheese-making efficiency. In this study, the combination of classical GWAS with the stepwise regression method and pleiotropy analysis was an efficient approach to discover the best combination of genetic variants underlying cheese-making traits. These SNPs, located within genes or in the confidence interval of 20 kb from a gene, can explain the highest proportion of genetic variance and could help to understand the role of the related genes and their co-associations on the studied traits. Through stepwise analysis, we selected two gene sets. The first significantly co-associated gene set, composed of 374 genes, could be useful for the design of a low-density SNP chip to generate information that could help to increase the efficiency of dairy sheep breeding programmes. The second selected gene set, composed of 4586 genes, might help to elucidate the role of the genes that influence cheese-making efficiency. This gene set also revealed how much of the average genetic variance of the 14 traits could be overestimated according to the markers selected for the corresponding analysis.

The functional enrichment analyses performed, based on multiple sources of information, enabled us to identify and classify the biological processes related to the two considered gene co-association networks. The first gene co-association network was composed of 374 genes, of which 55 were TF and CF. Among the TFs found, zinc-finger transcription, homeobox and ETS were the most common among families. These TFs are related to the control of the expression of multiple genes [33] involved in regulating the expression of target genes associated with cellular differentiation [34] and activating or repressing the transcription process [35]. For that reason, transcription and co-transcription factors were considered potential regulators of the network. Moreover, three transcription factors were located in the confidence intervals of the QTLs related to the traits under study: the *MECOM* gene related to cell differentiation and the regulation of transcription, the *ZNF250* gene associated with the regulation of transcription and the *ZFPM1* gene related to the cell morphogenesis process [11,36]. These genes were expressed in the sheep mammary gland during lactation [19], which supports their role in the synthesis of milk. All these genes could be considered functional candidates affecting milk and cheese-making traits in sheep. The enrichment analysis detailed 139 biological processes associated with protein metabolism pathways and 19 with fat metabolism pathways. Some of the genes that make up this first set (*CD44*, *ITPR1, PCSK2,* and *SLC20A2)* have shown a similar role in dairy cattle [15] and are detailed in Additional file 8.

The second gene co-association network consisted of 4586 genes, including 497 potential regulators of the network. This gene set includes two additional transcription factor families: bHLH (basic helix–loop–helix), one of the largest families of dimerising transcription factors, and HMG (high mobility group), which is involved in many biological processes, such as transcription, replication and recombination [37]. Among the new functions associated with transcription factors, the hormone-mediated signalling pathway should be highlighted due to its impact on milk production through the influence of corticotropin, prolactin and thyroid hormones [38,39,40,41,42]. The successive gene selection by the stepwise method has allowed extending the list of genes possibly involved in milk synthesis and cheese-making efficiency and has enabled significant biological processes associated with the gene set to be identified. The detailed significant functions were generally related to basal and essentially biological processes, but we should emphasise the homeostatic process, ion transport and cellular response to stress. Suárez-Vega et al. [19] also reported this last function as of significant relevance in the mammary gland, possibly due to the elevated rates of protein and fat synthesis faced by this organ during lactation. Finally, these results suggest that many general biological processes indirectly influence milk yield, composition and coagulation traits.

It is worth highlighting six of the genes gathered in the co-association network, which encode milk proteins or proteins involved in milk fat metabolism [19]. The *LALBA* gene, which encodes the whey protein α-lactalbumin, was reported to be strongly associated with protein and fat percentage in dairy sheep [11]. The *BTN1A1* gene, which encodes butyrophilin subfamily 1 member A1, and the *SLC27A6* gene, which encodes solute carrier family 27 member 6, were found to be associated in cows with lipid droplet formation and fatty acid uptake, respectively [43]. The perilipin-2 protein (encoded by the *PLIN2* gene) was found to be related to the packaging of triglycerides for secretion as milk lipids in the mammary gland [44]. Last, the *ACACA* gene, which encodes acetyl-coenzyme A carboxylase α, and the *SCD* gene, which encodes stearoyl-CoA desaturase, are related to fatty acid synthesis and desaturation [43]. The phospholipase A2-activating protein (*PLAA* gene), which is related to the protein phospholipid metabolic (GO:0006644) and prostaglandin metabolic processes (GO:0006693), and the acetyl-CoA acyltransferase 2 (*ACAA2* gene), which is involved in fatty acid catabolic process (GO:0009062), were also highlighted by the enrichment analysis carried out in a previous study of the transcriptome of the sheep mammary gland [19]. Moreover, Sanchez et al. [15] reported 62 genes included in this gene set (see Table S5) as possible functional candidates related to milk cheese-making properties. The effect of those genes on milk protein, milk fatty acid and milk mineral composition has also been supported in other studies [15,45,46,47]. Similarly, Cánovas et al. [48] reported three genes associated with citrate content in cow milk, coding for citrate synthase (encoded by *CS* gene), dihydrolipoamide dehydrogenase (*DLD* gene) and ATP citrate lyase (*ACLY* gene), which were also detailed in this gene set.

Furthermore, pleiotropy is defined as the presence of statistically significant associations of one marker with more than one trait [49]. Pleiotropic effects estimated for the co-transcription factors were higher than for the rest of the genes included in both gene networks which, together with the transcription factors, have been considered as potential regulators of the co-association networks presented in this study and therefore of the metabolic pathways related to milk and cheese-making traits. Apart from the transcription, co-transcription factors and coding genes, microRNAs (miRNAs) were also included in these gene networks; one miRNA was included in the first gene set selected (microRNA_125b-1), and 14 were included in the second selected gene set by stepwise analysis (see Table S2). The miRNAs are involved in the regulation of the expression of complementary messenger RNAs [50] and have a role in mammary gland development and lactation and lipid and fatty acid metabolism [51,52]. In addition, several unannotated genes have been found in both gene networks, which could codify for novel proteins or constitute functional noncoding RNAs. In addition, some genes potentially belonging to the zinc-finger transcription factor family have been found to be unclassified, as in AnimalTFDB 3.0 [29]. These findings reflect the incomplete annotation of the sheep genome, as previously suggested by Suárez-Vega et al. [19]. Therefore, it is important to consider that this incompleteness of the reference genome can complicate the interpretation of results from association studies.

To summarise, stepwise regression analysis is a computationally costly and exhaustive method for prioritising genes related to the analysed traits, which has enabled us to identify co-association networks composed of candidate genes and their potential regulators. In addition, the approach presented in this study has also allowed us to understand the co-association among the highlighted gene sets and their possible biological roles in milk and cheese traits in sheep. The co-association network composed of 374 genes may be suitable for the design of a low-density chip useful to predict an individual’s genetic potential for cheese-making efficiency. This approach would enable selection for these difficult-to-measure traits earlier in life compared with traditional selection methods [25]. Sheep milk is mostly transformed into cheese [53]. Therefore, it is important to implement genomic selection strategies for milk and cheese-making traits to improve cheese-making efficiency without causing negative effects for the selection for milk production and other functional traits of considerable interest for sheep breeders, such as SCC.

## 5. Conclusions

The combination of a stepwise regression forward selection analysis with classical GWAS, co-association network and enrichment analyses enabled us to identify two gene co-association networks. These networks were composed of potential functional candidate genes and gene regulators related to several biological processes that could have a direct or indirect effect on milk and cheese-making traits in sheep. In this study, we present a highly co-associated network composed of 374 genes, including transcription and co-transcription factors, with a potential influence on milk synthesis and cheese-making efficiency, which could be used to draft animals in future genomic selection programmes. The 374 SNPs within the confidence interval of a gene selected explained 76% of the average additive genetic variance of the 14 traits under study. In addition, an expanded highly co-associated network related to milk and cheese-making traits, identified and described in detail in this study, enhanced our understanding of the biological processes involved in milk synthesis and cheese-making efficiency. Increasing the number of animals could provide improved detection of variants significantly associated with the traits under study and therefore increase the accuracy of the genomic prediction. Further studies will be required to confirm the capability of the low-density panel of markers selected, based on our analyses, to predict the genetic potential of individuals for milk and cheese-making traits in independent populations, even in different breeds, to prove the versatility of the panel among dairy sheep breeds.

## Figures and Tables

**Table 1:** Results of the multifactorial ANOVA applied to the milk and cheese-making traits analysed in the present study.

Traits ^1^	Fixed factors ^2^				Coagulation ^3^	R-square (%) ^4^
	AGE_NB	NBL	DIM	FTD		
**Milk production traits**						
**MY**	***	*	*	***	***	34.52
**PP**	ns	ns	ns	***	ns	28.42
**FP**	ns	ns	ns	***	ns	20.57
**LP**	ns	ns	*	***	***	21.11
**DE**	ns	ns	ns	***	ns	22.17
**pH**	ns	ns	***	***	***	47.04
**SCC (log)**	ns	ns	ns	***	***	18.80
**Cheese-making traits**						
**A30**	*	ns	ns	***	na	26.81
**A60**	*	ns	ns	***	na	11.96
**RCT**	ns	ns	ns	***	na	10.61
**RCT/A60 (log)**	ns	ns	*	***	na	18.60
**K20 (log)**	ns	ns	ns	***	na	6.13
**ILCY**	ns	ns	ns	***	na	8.17
**ILDCY**	*	ns	*	***	na	11.52

^1^ MY, milk yield (kg); PP, protein percentage (%); FP, fat percentage (%); LP, lactose percentage (%); DE, milk dry extract percentage (%); Urea, urea content in milk (mg/mL); logSCC, logarithm of somatic cell count (cells/mL); A30, curd firmness at 30 min (mm); A60, curd firmness at 60 min (mm); RCT, rennet clotting time (min); logRCT/A60, logarithm of the ratio RCT/A60 (min/mm); logk20, logarithm of curd-firming time (min); ILCY, individual laboratory cheese yield (g/10 mL); ILCDY, individual laboratory dried curd yield (g/10 mL). ^2^ Significance level of the variance explained by the fixed effect included in the model for the different traits: [ ns (*p* > 0.05); * (*p* ≤ 0.05); ** (*p* ≤ 0.01); *** (*p* ≤ 0.001)]. Fixed factors: AGE_NB: ewe’s age at parturition combined with the number of births (18 levels), FTD is the flock test day (12 levels), and NBL is the number of born lambs (two levels, one or two lambs); DIM is the covariate of days in milk. ^3^ Significance level of the influence of milk traits on the coagulation success factor (na (not applicable)). ^4^ Percentage of variance explained by the model.

**Table 2:** Effect of the DIM covariable on the milk and cheese-making traits under investigation.

Traits ^1^	Days in Milk		
	Effect	SD	*p*-value
**Milk production traits**			
**MY**	0.0027	±0.0015	0.0401
**PP**	−0.0005	±0.0006	0.3998
**FP**	0.0024	±0.0014	0.0901
**LP**	−0.0007	±0.0003	0.0425
**TS**	0.0012	±0.0016	0.4655
**pH**	−0.0007	±0.0001	<0.0001
**SCC (log)**	−0.0011	±0.0008	0.1755
**Cheese-making traits**			
**A30**	0.0462	±0.0235	0.0495
**A60**	0.0288	±0.0247	0.2447
**RCT**	−0.0331	±0.0200	0.0986
**RCT/A60 (log)**	−0.0008	±0.0003	0.0289
**K20 (log)**	−0.0006	±0.0004	0.1724
**ILCY**	0.0018	±0.0013	0.1846
**ILDCY**	0.0013	±0.0005	0.0165

^1^ MY, milk yield (kg); PP, protein percentage (%); FP, fat percentage (%); LP, lactose percentage (%); DE, milk dry extract percentage (%); Urea, urea content in milk (mg/mL); logSCC, logarithm of somatic cell count (cells/mL); A30, curd firmness at 30 min (mm); A60, curd firmness at 60 min (mm); RCT, rennet-clotting time (min); logRCT/A60, logarithm of the ratio RCT/A60 (min/mm); logk20, logarithm of curd-firming time (min); ILCY, individual laboratory cheese yield (g/10 mL); ILCDY, individual laboratory dried curd yield (g/10 mL).

**Table 3:** Phenotypic means and genetic variances and heritabilities of each trait analysed in this dataset.

Traits ^1^	Mean	SD ^2^	σ2	SE^3^	h2	SE ^3^
**Milk production traits**						
**MY**	2.8885	1.0730	0.1999	0.0617	0.2501	0.0739
**PP**	5.0535	0.4639	0.0546	0.0128	0.3433	0.0746
**FP**	5.5558	1.0539	0.0366	0.0528	0.0402	0.0582
**LP**	5.1072	0.2396	0.0091	0.0035	0.1802	0.0668
**DE**	16.6060	1.2547	0.2101	0.0898	0.1656	0.0693
**pH**	6.7276	0.1336	0.0049	0.0011	0.3706	0.0751
**SCC (log)**	2.1896	0.6082	0.0170	0.0217	0.0479	0.0614
**Cheese-making traits**						
**A30**	29.7650	13.9750	48.5430	21.6810	0.2845	0.1230
**A60**	40.9590	11.2960	19.0450	8.1594	0.1658	0.0697
**RCT**	29.1540	10.5250	19.1840	6.5753	0.2255	0.0747
**RCT/A60 (log)**	-0.1535	0.2839	0.0130	0.0049	0.1956	0.0710
**K20 (log)**	0.5714	0.2227	0.0139	0.0040	0.3347	0.0905
**ILCY**	2.4938	0.4136	0.0519	0.0142	0.3366	0.0864
**ILDCY**	0.9724	0.1758	0.0065	0.0023	0.2593	0.0866

^1^ MY, milk yield (kg); PP, protein percentage (%); FP, fat percentage (%); LP, lactose percentage (%); DE, milk dry extract percentage (%); Urea, urea content in milk (mg/mL); logSCC, logarithm of somatic cell count (cells/mL); A30, curd firmness at 30 min (mm); A60, curd firmness at 60 min (mm); RCT, rennet clotting time (min); logRCT/A60, logarithm of the ratio RCT/A60 (min/mm); logk20, logarithm of curd-firming time (min); ILCY, individual laboratory cheese yield (g/10 mL); ILCDY, individual laboratory dried curd yield (g/10 mL). ^2^ SD: Standard deviations of the phenotypic values of each trait included in this study. ^3^ SE: Standard error of the genetic variance and heritability values represented in this table.

**Table 4:** Estimates of heritability (**diagonal in bold font**), phenotypic correlations (above the diagonal) and genomic (below the diagonal) correlations between the milk and cheese-making traits.

Traits	pH	RCT	logk20	A30	A60	ILCY	ILCDY	FP	PP	LP	DE	MY	logSCC	logRCT/A60
**pH**	**0.37 (0.07)**	0.47 (0.02)	0.37 (0.03)	−0.24 (0.04)	−0.44 (0.02)	0.03 (0.03)	−0.17 (0.03)	−0.09 (0.03)	−0.09 (0.03)	−0.16 (0.03)	−0.14 (0.03)	−0.11 (0.03)	0.30 (0.02)	0.50 (0.02)
**RCT**	0.63 (0.02)	**0.22 (0.07)**	0.68 (0.02)	−0.85 (0.02)	−0.61 (0.02)	0.03 (0.03)	−0.12 (0.03)	0.02 (0.03)	0.04 (0.03)	−0.04 (0.03)	0.02 (0.03)	0.03 (0.03)	0.15 (0.03)	0.91 (0.01)
**logk20**	0.57 (0.02)	0.92 (0.01)	**0.31 ** **(0.08)**	−0.68 (0.03)	−0.59 (0.02)	−0.05 (0.03)	−0.21 (0.03)	−0.02 (0.03)	−0.12 (0.03)	−0.01 (0.03)	−0.07 (0.03)	−0.05 (0.03)	0.12 (0.03)	0.74 (0.02)
**A30**	−0.44 (0.03)	−0.97 (0.01)	−0.86 (0.02)	**0.28 (0.09)**	0.34 (0.04)	0.05 (0.04)	0.14 (0.04)	−0.02 (0.04)	0.05 (0.04)	−0.02 (0.04)	0.02 (0.04)	0.05 (0.04)	−0.08 (0.04)	−0.75 (0.02)
**A60**	−0.76 (0.02)	−0.79 (0.02)	−0.95 (0.01)	0.91 (0.01)	**0.16 (0.06)**	0.01 (0.03)	0.22 (0.03)	0.07 (0.03)	0.13 (0.03)	0.02 (0.03)	0.19 (0.03)	−0.01 (0.03)	−0.07 (0.03)	−0.85 (0.01)
**ILCY**	−0.89 (0.01)	0.37 (0.03)	0.14 (0.03)	0.11 (0.04)	0.11 (0.03)	**0.33 (0.08)**	0.83 (0.01)	0.17 (0.03)	0.27 (0.03)	−0.06 (0.03)	0.23 (0.03)	−0.13 (0.03)	0.08 (0.03)	0.01 (0.03)
**ILCDY**	−0.36 (0.03)	0.23 (0.03)	−0.51 (0.02)	0.71 (0.03)	0.14 (0.03)	0.87 (0.01)	**0.26 (0.08)**	0.29 (0.03)	0.30 (0.03)	−0.14 (0.03)	0.27 (0.03)	−0.08 (0.03)	0.03 (0.03)	−0.19 (0.03)
**FP**	−0.50 (0.02)	0.36 (0.03)	0.66 (0.02)	−0.52 (0.03)	−0.99 (0.00)	0.99 (0.00)	0.99 (0.00)	**0.04 (0.05)**	0.27 (0.02)	−0.22 (0.03)	0.89 (0.01)	−0.03 (0.03)	0.04 (0.03)	−0.01 (0.03)
**PP**	−0.11 (0.03)	0.30 (0.03)	−0.11 (0.03)	0.70 (0.03)	0.16 (0.03)	0.60 (0.02)	0.74 (0.02)	0.65 (0.02)	**0.34 (0.06)**	−0.22 (0.03)	0.60 (0.02)	−0.30 (0.02)	0.18 (0.03)	−0.03 (0.03)
**LP**	−0.17 (0.03)	−0.23 (0.03)	−0.47 (0.03)	0.19 (0.04)	0.18 (0.03)	−0.40 (0.03)	−0.65 (0.02)	−0.41 (0.02)	−0.24 (0.03)	**0.18 (0.06)**	−0.08 (0.03)	0.21 (0.03)	−0.45 (0.02)	−0.04 (0.03)
**DE**	−0.16 (0.03)	0.22 (0.03)	0.16 (0.03)	−0.20 (0.04)	0.13 (0.03)	0.79 (0.02)	0.99 (0.00)	0.89 (0.01)	0.84 (0.01)	−0.11 (0.03)	**0.16 (0.06)**	−0.12 (0.03)	0.01 (0.03)	−0.03 (0.03)
**MY**	−0.27 (0.02)	−0.28 (0.03)	−0.22 (0.03)	−0.32 (0.04)	0.26 (0.03)	−0.34 (0.03)	−0.30 (0.03)	−0.42 (0.02)	−0.52 (0.02)	0.92 (0.01)	−0.50 (0.02)	**0.24 (0.07)**	−0.31 (0.02)	0.02 (0.03)
**logSCC**	0.58 (0.02)	0.12 (0.03)	0.26 (0.03)	0.32 (0.04)	0.83 (0.01)	0.48 (0.02)	0.36 (0.03)	−0.75 (0.02)	0.60 (0.02)	−0.73 (0.02)	−0.25 (0.03)	−0.97 (0.00)	**0.05 (0.05)**	0.13 (0.03)
**logRCT/A60**	0.70 (0.02)	0.97 (0.00)	0.96 (0.00)	−0.99 (0.00)	−0.92 (0.01)	0.19 (0.03)	0.70 (0.02)	0.13 (0.03)	0.15 (0.03)	−0.20 (0.03)	0.34 (0.03)	−0.25 (0.03)	0.32 (0.03)	**0.20 ** **(0.06)**

RCT, rennet clotting time; logk20, logarithm of curd-firming time; A30, curd firmness at 30 min; A60, curd firmness at 60 min; ILCY, laboratory cheese yield; ILCDY, individual laboratory dried curd yield; FP, fat percentage; PP, protein percentage; LP, lactose percentage; DE, milk dry extract; MY, milk yield; logSCC, logarithm of somatic cell count; logRCT/A60, logarithm of the ratio RCT/A60.

## Supplementary Materials

The following are available online at https://www.mdpi.com/2073-4425/11/7/715/s1, **Figure S1. Title: Distribution of genetic variance explained in each step of the stepwise analysis.** Description: This figure represents the distributions of the genetic variance explained by each round of the stepwise regression analysis. Per step, a total of 1000 randomly sampling of 50 new SNPs were performed to evaluate the genetic variance explained by each set of SNPs. The y-axis represents the different set of genes selected along with the stepwise analysis. The x-axis represents the distribution of the averages of the genetic variance explained for the 14 traits analysed. The red line (value 1) represents the total variance explained by all SNPs located in the confidence interval of a gene, being the total variance explained by the complete set of markers. The genetic variance explained situated over this value represent an overestimation, illustrated on a purple (zero genetic variance explained) to yellow (maximum variance explained) colour scale, **Figure S2. Title: Correlation between the off-diagonal elements of the GRM in each step of the stepwise procedure with the off-diagonal estimated by all the SNPs located in the confidence interval of a gene.** Description: This figure shows how the correlation among the off-diagonal elements of the GRMs generated by the stepwise analysis increase as the analysis progress. The x-axis represents the number of genes selected along with the stepwise analysis. The y-axis represents the correlation between the off-diagonal elements of the GRMs with the off-diagonal elements estimated by all the SNPs considered in the study, **Figure S3. Title: Pedigromics based on the two lists of genes sets used in the co-association gene network analysis**. Description: The figure represents the relationship (>0.2) between the samples included in this study obtained from the two gene-set selected through the stepwise analysis. Each node represents one animal from the population. The colour was represented based on the z-score, on a black to a white colour scale, with black being the highest value and white the lowest and the size of the nodes are based on the betweenness coefficient, the number of groups that these nodes connect, **Table S1. Title: Variants selected within the confidence interval of each gene that were used for the stepwise analysis.** Description: Worksheet providing the information of the 12,426 SNPs mapped within the confidence interval of a gene included in the stepwise regression analysis. Transcript column indicates whether these variants were identified within the transcriptome of the lactating mammary gland by Suárez-Vega et al. (19), **Table S2. Title: Results of the enrichment and association analysis of the two gene-sets selected to perform the co-association gene network analysis.** Description: Worksheet that provides the two lists of genes sets used in the co-association gene network analysis. For each gene, we describe the genomic position, the biological processes it was involved in, and the group of traits it was associated with in the association analysis, **Table S3. Title: Results of the biological process enrichment analysis.** Description of data: Worksheet providing the results of the 100 most significant biological processes identified through the enrichment analysis with the WebGestalt tool in both sets of genes selected by the gene network analysis, **Table S4. Title: List of genes selected through the stepwise analysis overlapping with reported QTLs related to the traits under study.** Description of data: Worksheet providing genes located within a region (±250kb) where a QTL was previously associated with the traits under study, based on the annotation of the SheepQTLdb, **Table S5. Title: List of genes included in the two gene-set co-association networks that are reported as possible functional candidates for milk cheese-making properties.** Description: Worksheet that provides the list of genes included in the gene-sets described in this study, previously reported as possible functional candidate involved in the milk cheese-making properties by Sanchez et al. (15). For each gene, we describe the genomic position, the biological processes it was involved, and the group of traits it was associated with.
