# Supplementary figures and images for: Gene Networks Driving Genetic Variation in Milk and Cheese-Making Traits of Spanish Assaf Sheep
Source: Genes (Basel). 2020 Jun 27;11(7):715. doi: 10.3390/genes11070715 (PMC7397207; doi:10.3390/genes11070715)

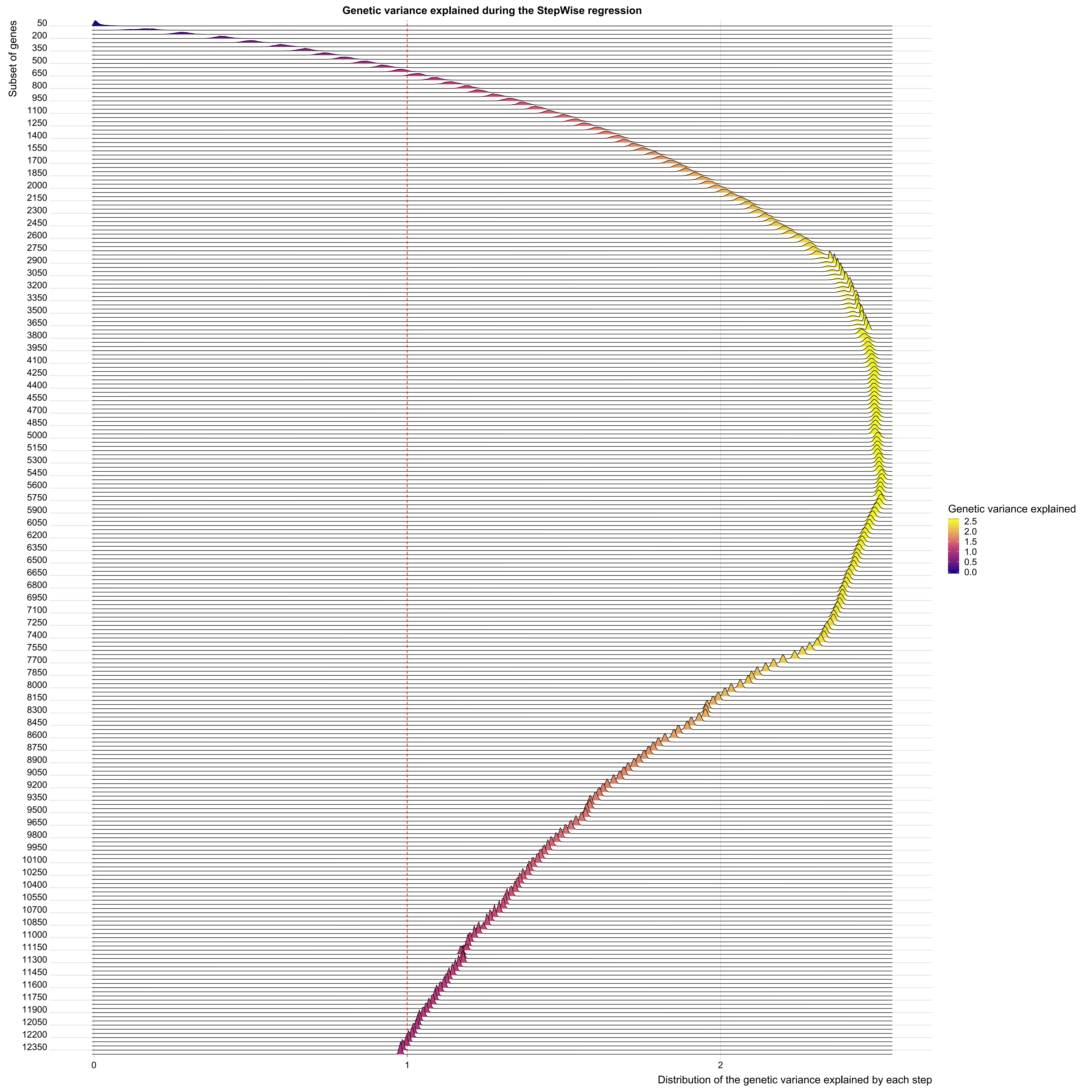

Supplement: Supplementary file 1 [file genes-11-00715-s001.zip › Figure S1.pdf]

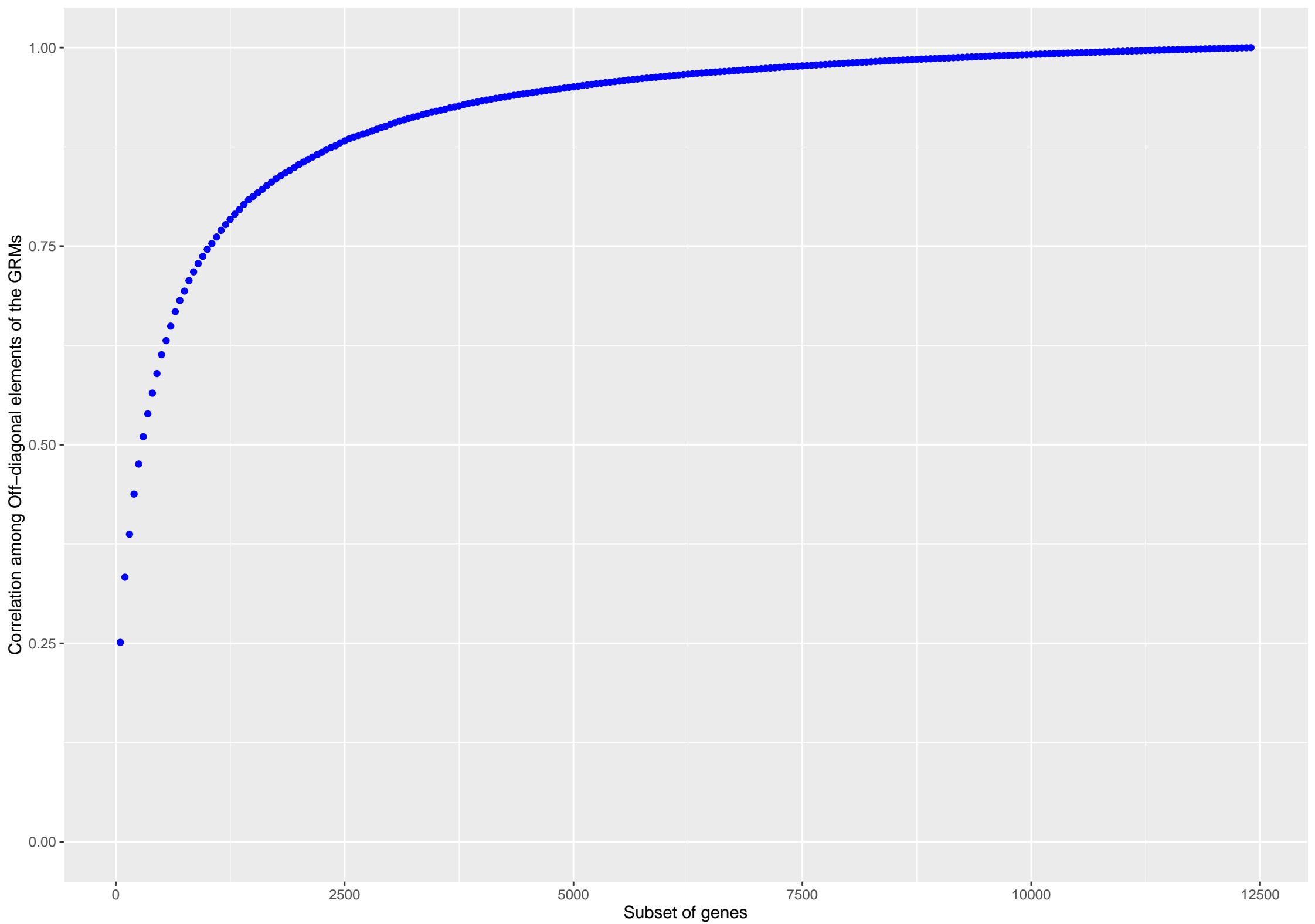

Supplement: Supplementary file 1 [file genes-11-00715-s001.zip › Figure S2.pdf]

Pedigromics: 374 genes.

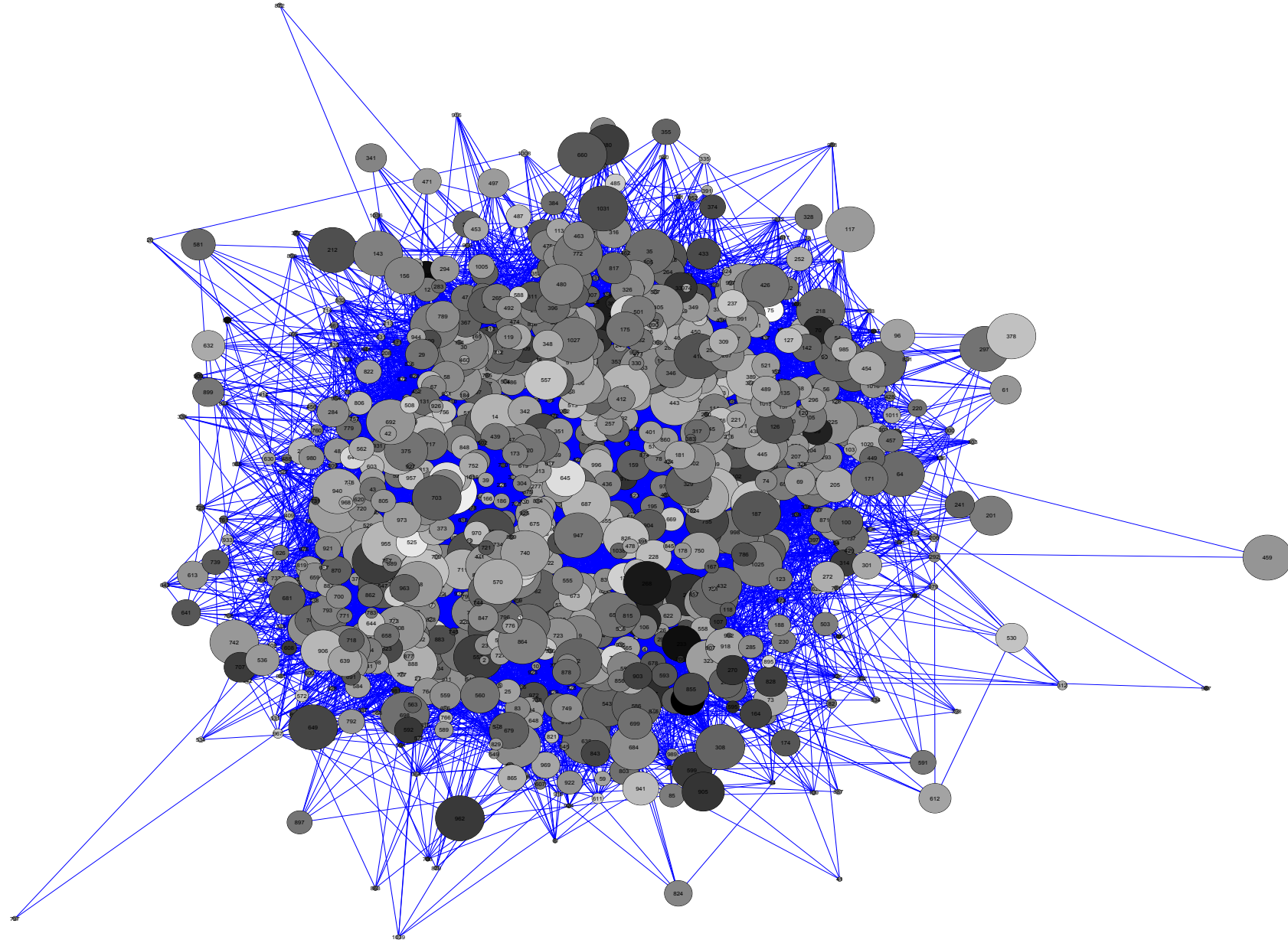

Pedigromics: 4586 genes.

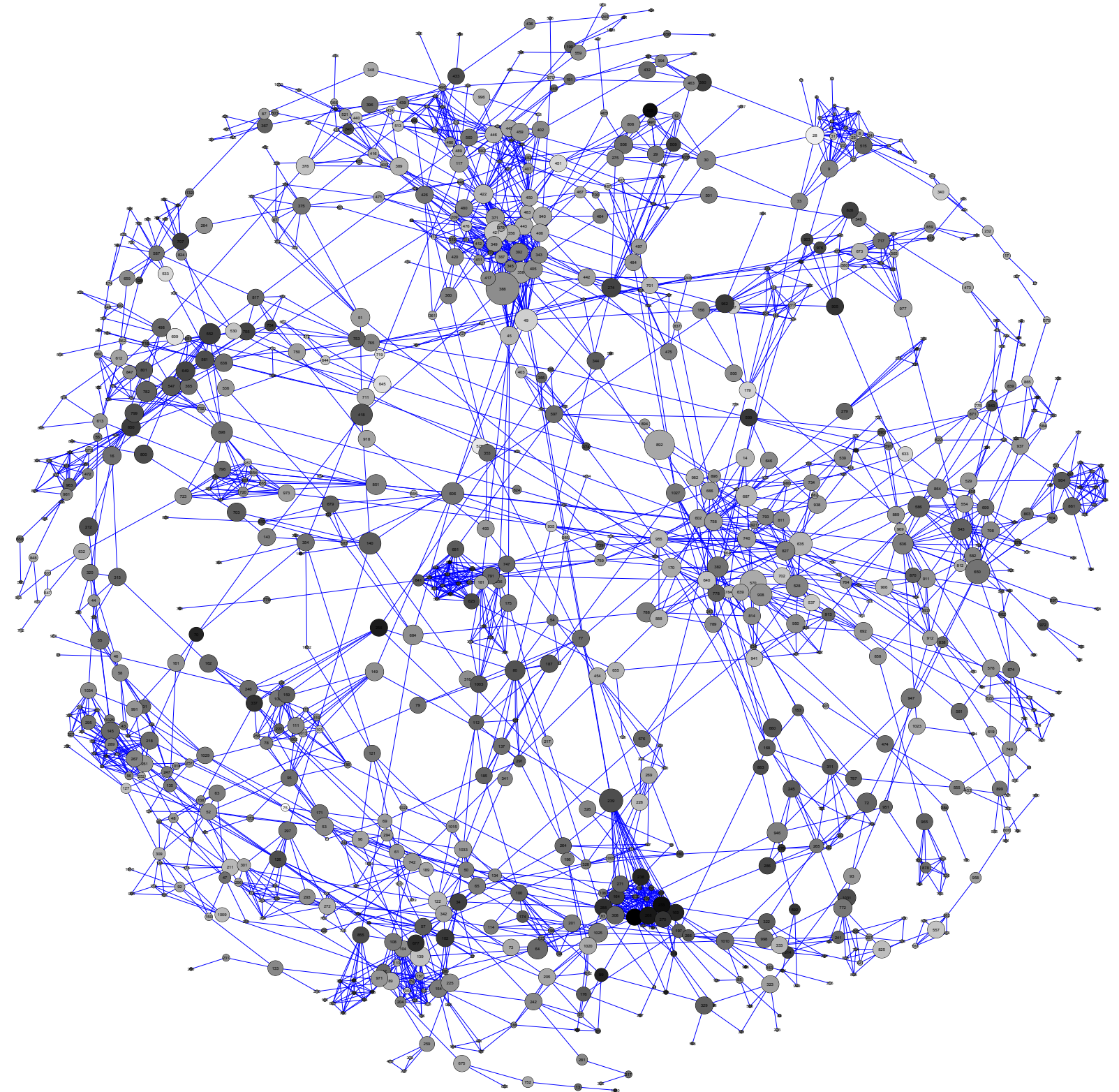

Supplement: Supplementary file 1 [file genes-11-00715-s001.zip › Figure S3.pdf]
